# Caudal ropivacaine and bupivacaine for postoperative analgesia in infants undergoing lower abdominal surgery

**DOI:** 10.12669/pjms.314.5432

**Published:** 2015

**Authors:** Surhan Ozer Cinar, Canan Tulay Isil, Sevtap Hekimoglu Sahin, Inci Paksoy

**Affiliations:** 1 Dr. Surhan Ozer Cinar, Associate Professor, Anesthesiology and Reanimation Clinic, Sisli Etfal Education and Research Hospital, Halaskargazi Cad. Etfal Sok. Sisli, Istanbul, Turkey; 2 Dr. Canan Tulay Isil, Specialist, Anesthesiology and Reanimation Clinic, Sisli Etfal Education and Research Hospital, Halaskargazi Cad. Etfal Sok. Sisli, Istanbul, Turkey; 3 Dr. Sevtap Hekimoglu Sahin, Associate Professor, Anesthesiology & Reanimation Department, Trakya University Faculty of Medicine. Edirne, Turkey; 4 Dr. Inci Paksoy, Specialist, Anesthesiology and Reanimation Clinic, Sisli Etfal Education and Research Hospital, Halaskargazi Cad. Etfal Sok. Sisli, Istanbul, Turkey

**Keywords:** Bupivacaine, Caudal analgesia, Pediatric anesthesia, Ropivacaine

## Abstract

**Objective:**

To compare the postoperative analgesic efficacy of ropivacaine 0.175% and bupivacaine 0.175% injected caudally into infants for lower abdominal surgery.

**Methods:**

Eighty infants, aged 3-12 months, ASA I-II scheduled to undergo lower abdominal surgery were randomly allocated to one of the two groups: Group R received 1ml.kg^-1^ 0.175% ropivacaine and Group B received 1ml.kg^-1^ 0.175% bupivacaine via caudal route. Postoperative analgesia, sedation and motor block were evaluated with modified objective pain scale, three-point scale and modified Bromage scale respectively. Postoperative measurements including mean arterial pressure (MAP), heart rate (HR), pain (OPS), sedation and motor block score were recorded for four hours in the postoperative recovery room. Parents were contacted by telephone after 24 hours to question duration of analgesia and side effects.

**Results:**

No significant differences were found among the groups in demographic data, MAP, HR, OPS and sedation scores during four hours postoperatively. The duration of analgesia was 527.5±150.62 minutes in Group R, 692.77±139.01 minutes in Group B (p=0.004). Twelve (30%) patients in Group R, 16 (40%) patients in groupB needed rescue analgesics (p=0.348). Rescue analgesics were administered (1 time/2 times) (9/3) (22.5/7.5%) in Group R and 16/0 (40/0%) in Group B, where no statistically significant difference was determined between the groups (p=0.071). Motor blockade was observed in 7 (17.5%) patients in Group R, and 8 (20%) patients in Group B (p=0.774).

**Conclusion:**

This study indicated, that a concentration of 0.175% ropivacaine and 0.175% bupivacaine administered to the infants via caudal route both provided effective and similar postoperative pain relief in infants, who underwent lower abdominal surgery.

## INTRODUCTION

In pediatric surgery, caudal anesthesia is commonly combined with general anesthesia since it suppresses neurohumoral response to surgery, accelerates recovery and enhances postoperative pain control. Because of decreased perioperative and postoperative analgesic requirements, which are the most important advantages of this technique, caudal anesthesia is commonly used in pediatric surgery for urological and lower abdominal procedures.^1^

Caudal anesthesia is a more simple regional block technique with fewer complications compared to other central block techniques, but there is still no ideal local anesthetic defined, which should be used for this technique. The most preferred local anesthetic in pediatrics is rasemic bupivacaine and it’s most important advantage is that it has a long duration of action. Ropivacaine is the analogous of bupivacaine having fewer cardiotoxic and neurotoxic side effects compared to bupivacaine.^2^,^3^ The analgesic effectiveness of ropivacaine is weaker than bupivacaine’s in adults.^4^ Whereas in infants, the analgesic effectiveness of ropivacaine is equal to that of bupivacaine, even in lower doses.^5^ The injection of local anesthetics into infants causes competitively decrease in dependent plasma proteins and increase of free local anesthetic amounts. Therefore, infants are more susceptible to local anesthetic toxicity.^6^,^7^ Previously a dosage of 0.175% ropivacaine administered via caudal route was proven to have sufficient analgesic effects without increased motor block frequency.^8^

The aim of this study was to compare postoperative analgesic efficacy of 0.175% ropivacaine and 0.175% bupivacaine injected caudally into infants, who undergo lower abdominal surgery.

## METHODS

Eighty infants, scheduled to undergo elective lower abdominal surgery in the pediatric surgery clinic, aged between 3-12 months, ASA I-II, were included in this prospective, randomized and single-blinded study after obtaining permission of local ethics committee (permission number: 26/09.02.2004) and obtaining parents’ informed consent.

Children with local infection, bleeding tendency, congenital spinal anomaly, neurologic diseases and having serious renal, hepatic, lung or cardiac diseases were excluded.

As premedication rectal or oral 0.05 mg.kg^-1^ midazolam was given to the children 30-40 minutes preoperatively. Routine monitoring was done with non-invasive blood pressure, electrocardiography and pulse oximetry measuring mean arterial pressure (MAP), heart rate (HR) and peripheral arterial oxygen saturation (SpO_2_). Anesthesia induction was done with sevoflurane concentrated to 4-6%. When the eyelash reflex was eliminated vascular access was achieved with 20-22G cannula. Orothracheal intubation was done with rocuronium bromide 0.6 mg.kg^-1^. Anesthesia was maintained with sevoflurane 2% concentrated in 50% N_2_O/O_2_.

Subjects were randomly divided into two groups with closed envelope method. All subjects were brought to decubitus position and single dosage of the medication was administered under sterile conditions with 22G epidural needle (Perifix pead®, Braun B, Meslungen, Germany) to caudal epidural space. Group R was given (n=40) 1ml.kg^-1^ 0.175% ropivacaine (Naropin® 2mg/mL 10mL ampule, AstraZeneca, Schaumburg, Germany) and Group B was given (n=40) 1ml.kg^-1^ 0.175% bupivacaine (Marcaine® 5mg 1 flakon, AstraZeneca, Kirklareli, Turkey) via caudal route in 20 seconds. Surgery was permitted 15 minutes after giving supine position to subjects. Since ethics committee required that anesthesiologists must be aware of the drugs administered to the children, the study was designed as single-blind. Caudal anesthesia was applied to all children by the same specialist.

Perioperatively sufficient analgesia was interpreted considering hemodynamic stability. Caudal analgesia was described as ineffective, when hemodynamical data rose 30% within the first 15 minutes after the block. Also an increase of 30% in these data during the postprocedural 45 minutes was evaluated as ineffective analgesia. Intravenous opioid (2 µg.kg^-1^ fentanyl) was injected to these patients and they were excluded from the study. Fluid maintainance was done with NaCl 0.2% in dextrose 5% in a dosage of 5 ml.kg^-1^.

The patients were transported to the postoperative recovery room after extubation. During the first four hours MAP, HR and SpO_2_ were recorded hourly by a monitor (Draeger Infinity Vista, Denvers, USA). Sedation depth, pain score, motor block and side effects were also recorded by a nurse, which was unaware of the given local anesthetic drug. The children were sent to the service after four hours observation, where they were discharged by the pediatric surgery clinic doctors and sent to their home. Patients’ parents were contacted by telephone after 24 hours and questioned about analgesic requirements and possible side effects.

Postoperative pain and analgesic requirements were evaluated with modified objective pain scale (OPS). Five criteria (crying, agitation, moves, posture and localization of place of pain) were assessed. Total score was between 0-10 and each criteria was given points between 0-2.^8^ Patients having OPS>4 were given rectal 30-40 mg.kg^-1^ paracetamol. Sedation was evaluated with the three point sedation score (1: awake, 2: sleepy woken up with verbal stimulant, 3: sleeping, woken up with physical stimulant). Motor block was assessed with modified Bromage scale (0= no motor block, 1=can move legs, 2=cannot move legs).^9^

After discharge from hospital; the family was asked to record how often and when rescue analgesics were used and if any side effects were noted. If analgesica was needed at home (OPS>4), oral 15 mg.kg^-1^ paracetamol (Calpol® 120mg 150mL, GlaxoSmithKline, Istanbul, Turkey) was suggested. The time interval from caudal block to the first analgesic consumption was considered as “analgesia time” and recorded.

Side effects as nausea-vomiting, sweating, pruritus, reddening and urine retention were recorded during the postoperative 24 hours. Children exhibiting nausea-vomiting were applied 0.1mg kg^-1^ (Metpamid® 5mg ampule, Sifar, Istanbul, Turkey) intravenously in the hospital. At home metoklopramid HCL was suggested as oral solution (Metpamid® oral 1mg 125 mL solution, Sifar, Istanbul, Turkey 1-2 scales).

For statistical evaluation, the results were expressed as mean±standard deviation (mean±SD) or in n (%). Compliance of data to normal distribution was examined with the single sampling Kolmogorov Smirnov test. In the comparison between the groups, Student t test was used for the variables indicating normal distribution and Mann-Whitney U test was used for those that do not indicate normal distribution. Chi-square test was used for comparison of categorical data. P<0.05 value was considered as statistically significant. The study was designed to be able to detect a 15% difference between study groups with regard to the number of patients with an absence (score 0) or presence (score 1–3) of motor blockade during the postoperative period. A power calculation based on these assumptions together with an α of 0.05 and a β of 0.8 resulted in the need for ≥25 patients in each treatment group.

## RESULTS

In this study, 80 subjects were included. Operation types and demographic features of children are shown in Table-I, where statistically no significant difference was determined (p>0.05).

**Table-I:** Demographic Characteristics and Operation Types.

	Group R (n=40)	Group B (n=40)	p
Age (month)	7.5±2.23 8(3-11)	7.05±2.60 9(3-12)	0.408†
Weight (kg)	8.37±2.62 8(4-12)	8.02±1.79 7(5-12)	0.488†
Gender M/F n(%)	34(85)/6(15)	35(87.5)/5(12.5)	0.745‡
ASA I/II n(%)	21(52.5)/19(47.5)	18(45)/22(55)	0.502‡
Operation time (min)	41.2±17.46 80(20-100)	52.25±25.56 105(20-125)	0.056†
Operation types			
Inguinal hernia n(%)	11(27.5)	8(20)	0.430‡
Hypospadias n(%)	14(35)	12(30)	0.633‡
Circumcision n(%)	15(37.5)	14(35)	0.816‡

M: Male, F: Female, ASA: American Society of Anesthesiology. p<0.05 is statistically significant,

Values are given as Mean±SD and Median (Range) or n(%),

† One-way ANOVA test.

‡ Chi-square test.

In the MAP and SpO_2_ values, that were monitored during the first postoperative four hours, no statistically significant difference was found within the group and between the groups (p>0.05).

In the postoperative first three hours, HR was statistically significant high in Group B compared to group R (p=0.036, p=0.001, p=0.012; Table-II).

**Table-II:** Postoperative MAP, HR and SpO_2_ Values.

		Group R (n=40)	Group B (n=40)	p
1^st^ hour	MAP	83.6±14.91	82.65±9.55	0.992†
	HR	125.35±17.59	133.82±17.99	0.036†
	SpO_2_	98.2±0.93	98.4±0.59	0.258†
2^nd^ hour	MAP	80.55±11.75	80.4±11.11	0.953†
	HR	125.6±15.63	138.07±17.96	0.001†
	SpO_2_	98.25±0.89	98.5±0.67	0.164†
3^rd^ hour	MAP	77.4±12.02	81.77±12.32	0.146†
	HR	126.05±16.78	135.35±15.68	0.012†
	SpO_2_	98.3±0.911	98.5±0.67	0.269†
4^th^ hour	MAP	79.85±13.24	83.85±10.87	0.144†
	HR	127.45±16.55	130.97±15.46	0.328†
	SpO_2_	98.35±0.80	98.5±0.75	0.390†

MAP: mean arterial pressure, HR: heart rate, SpO_2_: oxygen saturation.

p<0.05 is statistically significant.

Values are given as Mean±SD.

† One-way ANOVA test.

In the first four hours, sedation score was determined as second grade in 36 subjects of group R, and in 28 subjects of group B. Third grade sedation score was found in one subject of group R in the second hour and in one subject of group B in the first hour. No statistically significant difference was found among both groups (p>0.05, Table-III).

**Table-III:** Postoperative Sedation Scores.

	Group R (n=40)			Group B (n=40)			p
Sedation score	1	2	3	1	2	3	
1 hour	22(55)	18(45)	0	25(62.5)	14(35)	1(2.5)	0.429‡
2 hour	29(72.5)	10(25)	1(2.5)	32(80)	8(20)	0	0.504‡
3 hour	34(85)	6(15)	0	36(90)	4(10)	0	0.737‡
4 hour	38(95)	2(5)	0	38(95)	2(5)	0	1.000‡

Sedation score: 1: awake. 2: sleepy, woken up with verbal stimulant.

3: sleeping, hardly woken up with physical stimulant.

p<0.05 is statistically significant, Values are given as n(%),

‡ Chi-square test.

With regard to postoperative first four hour OPS, OPS was statistically found lower in first and third hours in bupivacaine group, no statistically significant difference was found in second and fourth hours (p=0.002, p=0.078, p=0.044, p=0.067; Table-IV). Analgesia time was recorded as mean±SD value 527.5±150.62 minutes in group R and mean±SD value 692.77±139.01 minutes in group B. In bupivacaine group, analgesia time was found statistically significantly long (p=0.004). Twelve (30%) subjects in group R and 14 (40%) subjects in Group B needed postoperative analgesics in the postoperative 24 hours (p=0.348). In group R (1 times/2 times) (9/3) (22.5/7.5%), in group B 16/0 (40/0%) additional analgesics were given and no statistically significant difference was found between the groups (p=0.071, Table-V).

**Table-IV:** Postoperative OPS Values.

		Group R (n=40)	Group B (n=40)	p
OPS value	1 hour	0.85±0.66	0.42±0.67	0.002†
	2 hour	0.65±0.66	0.42±0.67	0.078†
	3 hour	0.8±1.04	0.45±0.87	0.044†
	4 hour	0.45±0.67	0.25±0.63	0.067†

OPS: objective pain score.

p<0.05 is statistically significant.

Values are given as Mean±SD.

† One-way ANOVA test.

**Table-V:** Postoperative analgesia quality.

	Group R (n=40)	Group B (n=40)	p
Analgesia duration (min)	527.5±150.62	692.77±139.01	0.004†
Children requiring paracetamol n(%)	12(30)	16(40)	0.348‡
Sum of consumed analgesics n(%)(once/twice)	9(22.5)/3(7.5)	16(40)/0(0)	0.071‡

p<0.05 is statistically significant. Values are given as Mean±SD or n(%).

† One-way ANOVA test.

‡ Chi-square test.

First grade motor block was observed in 7 (17.5%) subjects in ropivacaine group and in 8 (20%) subjects in bupivacaine group. No statistically significant difference was found between groups in terms of motor block observance frequency and motor block grade (p=0.774, Table-VI).

**Table-VI:** Side Effects.

	Group R (n=40)	Group B (n=40)	p
Motor block	7(17.5)	8(20)	0.774‡
Nausea-vomiting	2(5)	2(5)	1.000‡
Itching	0	0	
Redness	0	0	
Urine retention	1(2.5)	0	0.314‡
Arrhythmia	0	1(2.5)	0.314‡

p<0.05 is statistically significant.

Values are given as n(%).

‡ Chi-square test.

When side effects were evaluated; postoperative nausea-vomiting was observed in two subjects in both groups, urine retention was observed in one subject of ropivacaine group and arrhythmia was observed in one subject of bupivacaine group. However, no statistical difference was found between the groups in terms of side effects (p>0.05, Table-VI).

## DISCUSSION

This study indicated that bupivacaine 0.175% and ropivacaine 0.175% used for caudal anesthesia in infants, who underwent lower abdominal surgery, both were effective and provided similar postoperative analgesia quality.

Bupivacaine is commonly used in pediatric patients for caudal block. However, its cardiotoxic side effect, although rarely observed, limits the usage of the medicine and causes the search for new less toxic medicines. Ropivacaine has less cardiotoxic effect compared to bupivacaine and its sensorial and motor effectiveness is superior to bupivacaine.^2^,^10^ The 0.2% preparation of ropivacaine can be used in all ages. However, pharmacodynamic responses may vary depending on age.^8^ Caudal analgesia effectiveness is directly related to volume and concentration of the medicine used for the block.^11^ It is indicated that ropivacaine 0.175% and 0.2% concentrations have similar analgesia time and quality in neonatal and infants.^8^ Bosenberg et al. obtained the same analgesic effect with ropivacaine 0.175% and 0.2% in a study conducted on children aged over one year and that ropivacaine 0.175% caused even less motor block development.^12^ In our study, ropivacaine having the concentration of 0.175% was used, because it is considered to have less side effects and similar analgesic effectiveness to bupivacaine.

Various scales are used for assessing the pain in the children. Brodmann’s pain score and OPS are preferred in this study, since they are practical and easy to use. Evaluation of acute pain in children may be less objective, when families are compared to health care team.^13^ However, families were involved for assessment of pain in this study, because they were closest to the children and could observe the children in the best way after discharge from the hospital.

Breschan et al. indicated that there is no difference between levobupivacaine, ropivacaine and bupivacaine in terms of analgesia time and quality and they found median analgesia time as 5.7 hours for ropivacaine and 5.35 hours for bupivacaine.^5^ Also, less motor block was observed in 0.2% ropivacaine and 0.2% levobupivacaine groups and the researcher recommended ropivacaine and levobupivacaine usage in daily surgery. Ivani et al. state that there is no difference in postoperative analgesia quality when 0.2% ropivacaine is compared to 0.25% bupivacaine and 0.25% levobupivacaine, and that slight reduction of early postoperative motor block is associated with the use of ropivacaine.^14^ Same researcher indicated in another study that similar postoperative analgesia is obtained when 0.2% ropivacaine and 0.2% levobupivacaine are compared and that there is no significant difference in motor block rates. Additionally, the median time of analgesia was found to be 6.3 hours in the ropivacaine group.^15^ In our study, motor block occured in 8 children in the bupivacaine group and in 7 children in the ropivacaine group. No total block, according to modified Bromage scale, was observed in any child. So, no statistically significant difference was established between the groups in terms of motor block development.

Khalil et al. used ropivacaine in five different concentrations in the children aged 1-7 years and indicated that with 0.1% ropivacaine more peroperative gas anesthetic and morphine was required.^16^ Luz et al. stated that singly administered 0.1% ropivacaine remained ineffective and that 0.2% ropivacaine was equivalent to 0.25% bupivacaine.^17^ Ingelmo et al. found median analgesia time of 0.2% ropivacaine, levobupivacaine and bupivacaine to be 2 hours and indicated that 0.2% ropivacaine is less effective than 0.2% levobupivacaine and bupivacaine during surgery.^1^ However, in our study, mean analgesia time was found to be 8.7 hours in the ropivacaine group and 11.5 hours in the bupivacaine group with statistically significant difference between the two groups. But, approximately 65% of the subjects, who participated in the study, did not need additional analgesics in the postoperative 24 hours. Furthermore, no difference was determined between the total consumption of paracetamol. Therefore, there was statistically no significant difference between the two groups in terms of analgesia quality despite analgesia time was longer in the bupivacaine group.

Postoperative vomiting (POV) occurred in the same number of subjects in both groups during the first two hours. Eberhart et al. defined four independent criteria for POV like following: duration of operation (≥30 min), presence of vomiting story, strabismus surgery and age of subject (≥3 years).^18^ Volatile anesthetic is also cause of POV especially in the early postoperative period until the first postoperative two hours.^19^ We considered that POV’s observed in our study were due to the fact that operation continued more than 30 minutes and volatile anesthetic was used. Urine retention was observed in only one subject in the ropivacaine group and arrhythmia developed in one subject in the bupivacaine group.^20^ This subject stayed monitored in hospital for postoperative 24 hours and was discharged in stable condition. Toxic reactions of local anesthetics are usually seen in organs like the heart and brain. It generally depends on overdose usage of medicine, application to intravascular or intraosseous area erroneously. No statistically significant difference was observed between two groups when side effects were compared.

## CONCLUSIONS

A concentration of both 0.175% bupivacaine and 0.175% ropivacaine administered caudally to children, who undewent lower abdominal surgery, ensured sufficient analgesic effect postoperatively. None of the two local anesthetics was superior to the other one regarding efficacy and side effects.
